# Barriers and facilitators to evidence based care of type 2 diabetes patients: experiences of general practitioners participating to a quality improvement program

**DOI:** 10.1186/1748-5908-4-41

**Published:** 2009-07-22

**Authors:** Geert Goderis, Liesbeth Borgermans, Chantal Mathieu, Carine Van Den Broeke, Karen Hannes, Jan Heyrman, Richard Grol

**Affiliations:** 1 Department of General Practice, Katholieke Universiteit, Leuven, Belgium; 2 Department of Endocrinology, University Hospitals, Leuven, Belgium; 3 Scientific Institute for the Quality of Healthcare, Radboud University, Nijmegen, the Netherlands

## Abstract

**Objective:**

To evaluate the barriers and facilitators to high-quality diabetes care as experienced by general practitioners (GPs) who participated in an 18-month quality improvement program (QIP). This QIP was implemented to promote compliance with international guidelines.

**Methods:**

Twenty out of the 120 participating GPs in the QIP underwent semi-structured interviews that focused on three questions: 'Which changes did you implement or did you observe in the quality of diabetes care during your participation in the QIP?' 'According to your experience, what induced these changes?' and 'What difficulties did you experience in making the changes?'

**Results:**

Most GPs reported that enhanced knowledge, improved motivation, and a greater sense of responsibility were the key factors that led to greater compliance with diabetes care guidelines and consequent improvements in diabetes care. Other factors were improved communication with patients and consulting specialists and reliance on diabetes nurse educators. Some GPs were reluctant to collaborate with specialists, and especially with diabetes educators and dieticians. Others blamed poor compliance with the guidelines on lack of time. Most interviewees reported that a considerable minority of patients were unwilling to change their lifestyles.

**Conclusion:**

Qualitative research nested in an experimental trial may clarify the improvements that a QIP may bring about in a general practice, provide insight into GPs' approach to diabetes care and reveal the program's limits. Implementation of a QIP encounters an array of cognitive, motivational, and relational obstacles that are embedded in a patient-healthcare provider relationship.

## Introduction

Landmark studies have demonstrated that intensive management of hyperglycemia, hyperlipidemia, and hypertension significantly reduces morbidity and mortality in patients with type 2 diabetes mellitus (T2DM) [1-9]. T2DM is a 'silent disease' until irreversible microvascular (*e.g*., nephropathy, retinopathy, diabetic foot) and/or macrovascular (*e.g*., myocardial infarction, stroke) complications become apparent. Prevention of these complications rests on timely institution of drug therapy by the prescribing physician, usually a general practitioner (GP), and the patient's compliance with the treatment regimen and willingness to make lifestyle changes. A proactive follow-up of diabetic patients is essential and should include foot examinations, blood and urine tests, and eye examination [10]. In addition, patients should be counseled about the dangers of diabetes and the importance of a healthy lifestyle, and impressed with the need for compliance with doctor's orders.

Unfortunately, many patients do not receive such level of care despite the availability of internationally-accepted treatment guidelines describing optimal management of patients with diabetes [11]. Optimal use of guidelines in general practice demands specific implementation strategies aiming at the reduction of barriers to high-quality care [12]. However, a clear understanding on how to overcome these barriers seems to be lacking [13-15], despite previous studies which outlined the obstacles that prevent GPs from following the guidelines [16-24]. Our study reports on 20 GPs who participated in an 18-month quality improvement program (QIP). The aim of this program was to improve diabetes-related patient outcomes through the implementation of evidence-based guideline recommendations. The different interventions of this QIP are described in the Appendix. The program resulted in significant improvements over time of HbA1c (-0.4%, CI 95% (-4;-3)), systolic blood pressure (-3 mmHg, CI 95% (-4;-1)) and LDL-C (-13 mg/dl, CI 95% (-15;-11)). However, results widely varied between participating GPs. Accordingly, we conducted a complementary, qualitative study (January to April 2008) nested in the controlled trial, to gain better insight into what changes the GPs had actually experienced. To fully understand these changes, we relied on an 'implementation model' based on the one described by Grol *et al*., 2004 [25-27].

## Methods

We conducted this qualitative research to acquire a better understanding of the barriers to high-quality diabetes care and into the mechanisms of change that eventually were induced by the QIP according to the experience of participating GPs. We opted for 'one-on-one' interviews in order to investigate the perceptions of the GPs about the QIP that essentially targeted the individual GP. We opted for semi-structured interviews in order to let the interviewees talk freely, as well as to deepen the interviewees' personal feelings about both the experienced barriers to high-quality care and facilitators of change.

To gain maximum information, the interviewees were randomly chosen from a stratified sample of participants according to clinical performance scores before and after the intervention. The clinical practices were divided in four strata relying on baseline performance (stronger versus weaker) and on the degree of improvement during the project (modest versus substantial). A researcher not involved in the interviews randomly chose five GPs within each stratum. If a selected GP refused to participate, the next GP on the list in that stratum was invited.

Interviewees and interviewers were blinded to the practice stratum at the time of the interview. Our design called for 20 interviews with *post-hoc *analysis and evaluation of data saturation. Plans were made for additional interviews if the data saturation criterion was not met. Three main questions were asked in the semi structured interviews: 'Which changes did you implement or did you observe in the quality of diabetes care during your participation in the QIP?' 'According to your experience, what induced these changes?' and 'What difficulties did you experience in making the changes?'

Subsequent discussions delved deeper into these topics by using an adaptation of 'reflective listening', a counseling technique that elicits a thorough disclosure of the interviewees thoughts and feelings [28]. It involves reflecting back to the interviewee what the interviewer believes was said in order to verify or clarify the interviewee's statements, and encourages interviewees to continue elaborating their views. In our interviews, not only were the assertions reflected back, the interviewees were also actively confronted with eventual inconsistencies in their answers. Throughout, the interviewers provided reassurance by intonation and body language in order to disclose the very personal feelings and experiences of the interviewees.

The interviews took 30 to 45 minutes and were conducted individually by two experienced researchers (GG and LBO), one a practicing GP and the other a community nurse specializing in health care consultancy. All interviews were taped and transcribed.

Before analyzing the transcripts, we discussed the analytical method to use. We decided to categorize the items by theory-based deduction using the 'implementation model' (Grol *et al*., 2004). We chose this model because it is based on a comprehensive overview of theories on implementation and behavioral change. These theories relate to the individual's cognitive, educational, and motivational attributes, as well as social, organizational, and economic factors. This model also reflects the basic structure of the interviews: barriers and facilitators of guideline implementation are well-described. As such, this model allows for deductive coding and categorizing of the items according to the level of action. After a first discussion round, we reached consensus to categorize the items in three levels: individual GP, individual patient, and social interaction, context, and organization. Items were divided into 'barriers to high-quality diabetes care' and 'factors facilitating change'. Barriers at the individual level were further categorized into subcategories of 'knowledge', 'awareness', 'attitude and motivation', 'routine' and 'others'. All transcripts were re-read when necessary and independently analyzed by GG and LBO to ensure reliability of the data. Transcripts were manually coded and the items were categorized using MicroSoft Excel spreadsheets. Differences in coding were discussed and final decisions on items and categories were based on a consensus between the two interviewers.

## Results

Two GPs refused to participate in the interview and were replaced by the GP next in line. In a post-hoc analysis, we found that few new themes were emerging after about 17 interviews, making it unnecessary to continue the interviewing after the 20 initially planned interviews. Table 1 shows the main characteristics of the interviewees that were felt to be typical of all 120 participants in the QIP. Table 2 shows the results of itemization that was obtained in commons consensus by the two researchers.

**Table 1:** Principal characteristics of participating GPs

	**S1** (N = 5)	**S2** (N = 5)	**S3** (N = 5)	**S4** (N = 5)	**All interviewees **(N = 20)	**All participants **(N = 120)
Mean age (years)	46	45	48	36	44	44
Females (N)	1	1	1	3	6	45%
Workplace						
Solo practice (N)	3	3	0	1	7	38%
Two man practice (N)	0	2	3	1	6	32%
Group practice (N)	2	0	2	3	7	30%

S1 = Stratum of GPs with weaker baseline performance and modest improvement during the QIP

S2 = Stratum of GPs with weaker baseline performance and substantial improvement during the QIP.

S3 = Stratum of GPs with stronger baseline performance and modest improvement during the QIP.

S4 = Stratum of GPs with stronger baseline performance and substantial improvement during the QIP.

**Table 2:** Coded categories and themes

**Perceived barriers to optimal diabetes care**		
**Level**	**Factor**	**Item**

Physician	Lack of knowledge on	- global cardiovascular treatment beyond glycemic control - insulin therapy
	Lack of awareness regarding	- personal practice performance ('blind spots') - need to reach treatment targets and regular follow-up
	Attitude and motivation	- laxity regarding treatment targets and timely follow-up - attitude to polypharmacy - skepticism regarding evidence-based treatment, top-down quality improvement projects and shared care collaboration
Patient	Practice organization	- lack of scheduled visits, lack of planned follow-up, lack of support staff
	Lack of knowledge on	- insight regarding complications, significance of HbA1c
	Lack of awareness regarding	- personal dietary patterns - personal health status (HbA1c, blood pressure, cholesterol)
	Attitude and motivation	- fear of insulin treatment - lack of motivation for follow-up or to change lifestyle
	Routine behavior	- maintaining lifestyle change very difficult - adhering to planned follow-up visits is difficult
Context and organization	Age and co-morbidity	- too strict control can be dangerous in older patients - immobility hampers physical exercise and shared care referral
	Relationships	- between GPs and patients (inertia to change) - competition between specialists and GPs
	Lack of teamwork	- Need for clear description of each provider's duties and responsibilities - Need for identical messages to the patients from all health care providers
	Financial barriers	- out-of-pocket payments for education, dietary advice and HBGM material - skewed reimbursement of HBGM material - fee for service: this system doesn't motivate GPs to deliver high-quality care

**Perceived change facilitators**		

**Level of impact**		**Item**

Physician		Treatment protocol and post-graduate education; Benchmarking feedback Case coaching; Timely data collection Increased contact and communication with peers in other disciplines Participation in team meetings Attitude change on the part of specialists
Patient		Nurse educator and IDCT working as a team Free services and free materials Identical messages from different sources (GP, specialist, educator, television Attitude change on the part of the GP
Context and organization		Role redesign and reassignment of responsibilities Serial removal of barriers Task relief

HBGM = Home Blood Glucose Monitoring; IDCT = Interdisciplinary Diabetes Care Team (endocrinologist, nurse educator, dietician) installed at the primary care level

All but four of the GPs confirmed the importance of improved adherence to the evidence-based guidelines. The four GPs who did not experience improved adherence belonged to a stratum with a stronger baseline performance, and three of them also belonged to the stratum with weaker improvement during the project. Three of them revealed that they had previously followed an intensive course on diabetes management. The fourth GP is still collaborating with the medical faculty of the university. Most interviewees also reported improvements in follow-up procedures, evidence-based drug prescription practices, and referral rates. The more frequent follow-up visits included regular blood monitoring and general screening for complications. Several GPs mentioned better recordkeeping.

Implementation of evidence-based treatment was evident in more timely adjustments in therapy if target criteria fell short, and in greater attention to cardiovascular risk factors, above and beyond conventional glycemic control. Finally, more patients were treated with insulin.

Some interviewees reorganized their practices to better comply with the guidelines. Others instituted regularly scheduled office visits, and some split the visits into two parts: one part dedicated to routine follow-up and the other to discussions of treatment and lifestyle. The interviewees noted better medication compliance and improved adherence to follow-up schedules by the patients.

### Barriers to high-quality diabetes care and factors facilitating change

Our analysis showed that a first barrier to successful diabetes care was GPs inadequate knowledge how to manage insulin therapy and cardiovascular risk.

'My attitude about insulin therapy onset has changed. Before the start of the project, I tried too long oral anti diabetics, but the courses have changed my attitude. I became confident in starting insulin therapy, whereas before I would never initiate insulin therapy. (12-S3)

A second barrier was the GPs' lack of awareness of their own performance because of 'blind spots'.

'Such a project with follow-up is important because it obliges you to question yourself. I thought my patients were reasonably well controlled, but the QIP – especially the feedback – makes you confront your problems and weaknesses.' (3, S1)

Several interviewees also affirmed that before the start of the project they did not truly understand the importance of attaining clinical targets and regular follow-ups.

'The constant support and the organized courses made the difference. The protocol map, which has become a reference work, also contributed a lot. Because of the feedback, I became aware that my performance on lipid-lowering therapy was not good. This, together with information on vascular pathology as a major problem in diabetes, made me change my attitude. I have begun to prescribe more statins.' (10-S3)

A third barrier, expressed by several interviewees, was the presence of skepticism about evidence-based treatment and of collaborative care, and their concerns about losing control and sanctions that may result from diabetes care improvement plans.

'I do everything myself. I find it difficult to work in a team, and I am rather skeptical about the 'soft sector' (psychologists, educators...)' (11-S3)

'Policymakers should use such programs for positive motivation. They should not connect results with negative implications (e.g., loss of accreditation).' (15-S3)

Some GPs considered evidence-based medicine (EBM) only as background information describing the ideal situation to strive for, but not as a stringent, compulsory framework.

'Paper is no reality. EBM is only a supportsupport tool, but can never be an impsosed framework.' (3-S1)

One GP admitted that he had worked according to a fundamentally different paradigm closer to alternative medicine. From this viewpoint he disagreed with the guideline on many aspects, such as the importance that was given to lipid control.

'Evidence-based medicine is a relative term...something might be evidence-based, but I have in mind other parameters that are much more important. In my alternative point of view, I do not care a lot about cholesterol, for example.' (7-S2)

Some GPs admitted being lax and several indicated that lack of time – because of suboptimal practice management – prevented them from providing good quality care.

'I admit that I was lax before, but have changed during the project. Some patients were incredibly surprised that finally they were getting good care.' (7-S2)

'I didn't observe major behavioral changes in most patients, but this may be associated with my own passive attitude. I made no changes in my organization of care and I did not spend enough time at it.' (16-S4)

Several GPs also questioned the feasibility and desirability of implementing these guidelines in an older diabetes population.

'Many of my patients are older than 80. I will not forbid them to eat a piece of cake. Indeed, my own attitude towards elderly people is a little bit more loose.' (4-S2)

'The recommendations on weight loss and physical activity are useless for a lot of elderly people who are too ill or immobile to follow them.' (3-S1)

Factors conducive to good care were also discussed. The consensus was that transparent treatment protocols and tailored post-graduate courses would go a long way in overcoming knowledge gaps. Benchmarking feedback confronted the GPs with their blind spots and weaknesses, and increased their awareness of shortcomings in their case management habits. Case coaching was identified as an important innovation in improving 'knowledge on the spot', especially in initiating and adapting insulin therapy.

'The extra coaching was unique to this project and functioned like clockwork. You only had to make a phone call – that is very comforting to a GP.' (12-S3)

Several GPs confirmed that the three-month data collection exercise encouraged regular recordkeeping and a structured approach to patient follow-up.

'The imposed recordkeeping of patient data put me under some pressure. Imposing a structure helps you handle your job more systematically. Since the project has stopped, this disciplined approach is beginning to wane again.' (1-S2)

Many GPs also felt that care was compromised by the patients' insufficient understanding of diabetes, lack of awareness of serious complications, and of the importance lifestyle changes. Fear of insulin therapy ('fear of the needle') was also mentioned. However, these barriers were perceived as something that could be overcome by education, especially when provided by well-trained nurse educators.

'The big change is the availability of the nurse educator... She really took the time to explain the problem of diabetes. People have a better understanding of what HbA1c is...people are afraid of needle sticks and this fear has decreased because of the project, thanks to the nurse educator.' (2-S2)

GPs also described the synergistic effect of several healthcare workers delivering the same message in inducing a sudden change in attitude.

'If three professionals give the same message and if, moreover, patients receive the same message by television, and then a sudden change can occur.' (8-S1)

There was consensus that patients' attitudes and lack of motivation are major barriers to implementing evidence-based treatment, especially when it involved a change in lifestyle.

'Physical activity and weight control remain the main problems. The motivation to change lifestyle habits is often completely absent. Some patients deny the problem: 'I don't eat very much'. (9-S2)

Finally, GPs felt that about one-third of the patients would be uncooperative no matter what changes were proposed, and most GPs agreed that changing entrenched lifestyle habits was difficult for most patients to achieve, whatever their initial motivation. For the most part, any such changes would be small and temporary.

'A minority – about 30% – doesn't want to hear anything. They won't even go to see the nurse educator. Another 30% are somewhat motivated, but not too much, and the remaining 30% really cooperate. The added value of the project, probably, applies only to patients who are motivated and who can get motivated.' (2-S2)

GPs also mentioned social, organizational, and legal barriers and facilitating factors. The interaction between a GP and his or her patients, especially when it concerns a long-term relationship, can itself hamper the transition to high-quality diabetes care. Several GPs described how patients were accustomed to certain situations and habits of their GPs, *e.g*., a limited use of drugs. They did not always understand or appreciate the sudden change in their GP's attitude; this led to tensions in some cases and loss of contact in others.

I have started prescribing lipid-lowering drugs relatively recently. Before the project, I was rather reluctant to prescribe medications and my patients were not accustomed to my new attitude. So, I had to take a gradual approach.' (10-S3)

'Previously, some patients probably consulted me because I was easygoing. Since my participation in the project, I've pushed them more and so I lost two patients. They frankly told me 'We're leaving because you exaggerate things. What's the matter with you?' But patients and physicians must evolve together, although at a moderate pace.' (7-S2)

However, the project mitigated such unfortunate instances through counseling sessions involving the GPs, patients and nurse educators. The net effect was a strengthening of the physician-patient relationship and a motivational boost to the latter.

'Diabetes patients themselves feel much more appreciated; because of that, the link between us and our patients has strengthened.' (17-S4)

Most GPs held that a lack of a clear delineation of responsibilities leads to competition between the GP and the specialist, with the latter being perceived as holding the upper hand. This competition is reinforced by the skewed reimbursement schemes in Belgium in favor of the specialist concerning patient education and home blood glucose monitoring (HBGM) kits. This skewed situation was considered as an important factor that prevents many GPs from commencing timely insulin therapy.

'Specialists gain too much control of referred patients and often exclude GPs from direct patient care. This is especially true of patients on insulin who get free instructions and monitoring kits at the diabetes centers, unlike patients in primary care. So, it's nearly impossible for GPs to hold on to patients on insulin.' (1-S2)

The QIP redefined the GP as a central 'manager' with explicit responsibilities for the care for patients with diabetes.

'To summarize this project: we started with a good protocol and established better channels of communication between primary and specialist care....The delineation of responsibilities and degree of familiarity among the partners were very important in making it easier to me to refer more patients.' (14-S1)

This was much appreciated by the interviewees. It reinforced the GPs' feeling of recognition, boosted self-esteem, promoted a greater sense of responsibility, and improved their professional relationships with specialists.

'The project did not merely create the illusion that the GP was pivotal in diabetes care, he or she actually became the central figure and this fact increased their job satisfaction....This only became possible because of an attitude change on the part of the endocrinologists. Now they say 'you GPs have to do the job, but call me when necessary.' This is a big change from the usual 'let us do our work; after all we are the specialists and you may help a little bit'. We collaborate as one team – there's mutual support! We're on the same wavelength and feel we work together toward the same objectives.' (13-S4)

Many GPs regarded the role of the nurse educator as complementary to their own and, feeling that they themselves lacked the requisite skills and time, were relieved to relinquish patient education to them.

'I prefer to have the nurse educator bring up insulin therapy before I get to it....After 30 years in general practice, I'm somewhat hesitant to get into a protracted struggle with patients to try to convince them of the need for insulin. 'If you're not interested, so be it,' I think by myself. The nurse educator is an invaluable asset in such cases.' (8-S1)

One GP felt that the Belgian fee-for-service scheme was an important impediment to the delivery of quality care, explaining that a pay-for-performance system would be a better motivator. In addition, direct payment by patients was also seen as a significant factor that discouraged patient referrals and HBGM necessary to evaluate insulin therapy.

## Discussion

Previous studies have disclosed a significant gap between the quality of diabetes care commonly encountered and recommended evidence-based guidelines [14]. To date, most research on barriers to and facilitators of high-quality care has been done before the start of improvement programs. Our study was based on interviews with GPs who actually participated in a project aimed at optimizing diabetes care. This approach, combined with the 'reflective listening' technique, elicited disclosure of very personal feelings and experiences related to changes in performance. As such, qualitative research nested in an experimental trial may clarify the improvements that a QIP brings about in a general practice.

The primary finding was that the project accomplished more than merely improving the quality of care. It also impacted the emotional and motivational status of the GPs. Previous focus group-based research had revealed that GPs working in the 'usual' setting in our country felt frustrated, partly because they felt inferior to specialists [29]. We showed that role-redesign and delineation of responsibilities *vis-à-vis *the specialists enhanced a GP's self-esteem and sense of responsibility. All interviewees were unanimous that this project was very beneficial because it added value to their jobs, even though some were concerned that QIPs could have manipulative ends or lead to sanctions.

Second, most of the GPs reported a major improvement in their diabetes care. According to the theory of planned behavior, decisions are made according to personal models and beliefs about the changes about to be made, and the perceived benefits and risks associated with them [30]. Several GPs indicated that the changes resulted from a conscious decision based on interconnected key elements during the quality improvement process. Reported key elements were the need to keep up with knowledge, the increased awareness that their practice needs improvement, and that their attitude needs adjustment. The GPs also observed attitudinal changes in their patients, *e.g*., better adherence to drug regimens and follow-up visits.

Third, a multifaceted QIP may evoke complex changes that go beyond individual physicians and patients, because they form an interconnected and interdependent social continuum. The GPs described cases in which joint and coherent actions of several health workers effected a change in a patient's attitude where a solitary GP failed. The QIP facilitated patient referrals to the nurse educator, despite certain resistance on the part of some patients or physicians. The nurse educator, in turn, contributed to patient care by ensuring follow-ups, providing information on insulin therapy and health lifestyles, and performing complementary examinations, *i.e*., carrying out functions for which the GP lacked time or did not possess adequate skills or motivation. This task delegation allowed the GPs both to sustain their ongoing relationship with the patients and to concentrate the efforts on their essential tasks, which are the medical management and follow-up of diabetes.

Finally, the QIP also altered interpersonal relationships. Most GPs confirmed that the QIP strengthened their relationships with their patients and improved communications with specialists and other healthcare providers. They also perceived a change in attitude on the part of the endocrinologists toward them, which markedly enhanced the GPs' motivation and sense of responsibility. These findings substantiated various theories and research findings that a positive relationship among healthcare providers is an important component of high-quality patient care [31,32].

Nevertheless, limitations of the QIP were also described. First, according to the interviewees, a significant minority of patients remained refractory to change, with many refusing to see a nurse educator. Most patients found it difficult to change their lifestyle, and even in the case of motivated individuals the changes were often minimal and temporary. These findings confirm previous findings that sustainable lifestyle changes are hard to implement in clinician-centered models of patient education [18,33-35]. Moreover, these models are labour- and resource-intensive [36] and traditionally put the emphasis on imparting knowledge [37]. Yet, in even the most successful trials of face-to-face education, many participants are not willing or able to attend the sessions [38,39]. Therefore, ongoing research evaluates the effect of new models that are based on peer support. These models put the emphasis on coping with illness, rather than managing it [40]. Peer support seeks to build on the strengths, knowledge and experience that peers can offer. Greenhalgh *et al*. has tested the effect of a narrative method (a person telling a story) versus conventional nurse-led education in a minority ethnic group of people with diabetes [40]. The results show that unstructured storytelling is associated with improvement of patients' enablement and comparable changes in biomedical markers. Other self-management programs evaluate the effect of other peer support interventions, like telephone counseling or web-based peer support. Future QIPs may incorporate peer support interventions replacing or complementing the traditional clinician-centered patient education interventions.

At GP-level, four interviewees affirmed not having experienced a major impact of the QIP on their quality of care. In fact, they experienced the QIP somehow as superfluous because they already paid special attention to evidence-based diabetes care before the start of the project. The study also revealed that some GPs were reluctant on to reorganize their practices to comply with the project's requirements, or even to find the time for efficient patient follow-up. Accordingly, future QIPs should specifically address such issues. Moreover, while the project was indeed able to induce a change in attitude with regard to medical diabetes treatment, some other deeply rooted attitudes were more difficult to change. For example, several GPs asserted that nurse educators and other personnel in the so-called 'soft sector' are of little value in good diabetes care. Collaborative shared care with specialists also remains a concern, despite the improvement that was observed during the project. One GP reported persistent problems with one local endocrinologist who was blamed for his disdainful attitude to general practice. Other GPs described minor remaining difficulties with endocrinologists despite overall satisfaction with the arrangements. These findings complement previously reported difficulties in collaborative shared care. One of the major reported issues about shared care is the problem of suboptimal communication between the involved providers [41]. This problem is associated with discontinuity in care and lower quality of care [42]. Other problems are related to lack of clear division of tasks and responsibilities between the involved providers, eventually leading to overlap and competing interests [29,43]. Despite these problems, we think that shared care is necessary to guarantee high-quality diabetes care because the management of this disease is too complex and too broad to have it provided by one person. However, the aforementioned problems are a real point of concern. Moreover, as our research shows, providers are not always willing to collaborate. Thus QIPs should pay special attention to eventual relational problems, to communication issues and to the distribution of rights, responsibilities and tasks between patients, GPs, nurse educators and specialists.

The role of EBM in daily practice remains a point of controversy. While many GPs accepted the existing guidelines, some did not. Some GPs fundamentally disagreed with EBM. Others accepted EBM as background support, but were afraid that EBM would be used to impose coercive instructions for daily practice. Several GPs questioned the feasibility and desirability of the American Diabetes Association guideline-based recommendations in the elderly or immobile people. Indeed, elderly patients are particularly sensitive to the adverse effects of drugs and polypharmacy, putting constraints on the classic diabetes treatment. In particular, hypoglycemia is an important topic in the diabetes treatment of elderly people. Recent studies [44,45] clearly indicate that hypoglycemia may be a contributing factor to morbidity and mortality in older patients. As such, strict adherence to guidelines for younger patients could be deleterious for the frail elderly [46]. Geriatric guidelines on the management of type 2 diabetes accentuate that treatment should be holistic, targeting all important aspects of the geriatric patients with priorities in the treatment scheme. Diabetes-related targets should be individually adapted to the frail patients with special attention to avoidance of side effects [47-49].

This qualitative research presents some limitations. A first possible bias concerns the researchers who conducted the interviews. They were previously involved in the QIP, and thus they are known by the interviewees as promoters of this program. As a consequence, GPs in disaccord with some issues of the QIP-process may have been discouraged to mention them. The GP cohort selected for the study represented an additional limitation. The participants were part of a larger sample of volunteer GPs who were particularly interested in the project. This selection bias may well be reflected in their answers. In order to generate a broad spectrum of answers regarding barriers to change, we employed a targeted sampling procedure that took into account the performance of the GP's practice. Only their subjective feelings and views are covered here, although a more balanced picture would have emerged if a joint patient-provider perspective had been offered. It remains for future research to include interviews with patients and, perhaps, employ mixed focus groups, and audio- or video-record observations of the clinician-patient encounters. However, despite the possible bias, we feel this qualitative study has provided a very balanced overview of the QIP's strengths and weaknesses, and validated the quantitative findings that had been obtained.

## Implications

Previous research revealed numerous barriers to high-quality diabetes care at the level of provider, patient, and healthcare organization. However, most of this research was done outside the context of quality improvement. Our research reveals the viewpoints of physicians who experienced a quality improvement process and it allows for evaluating the complex interactions between barriers and facilitators during this process. It has become obvious that implementation of a QIP encounters an array of cognitive, motivational, and relational barriers that are embedded in a patient-healthcare provider relationship. As their success may depend on overcoming key barriers, QIPs should incorporate mechanisms to actively detect and overcome these barriers or to cope with them. Moreover, several barriers appear to be interdependent, developing several 'chains of barriers'. This phenomenon may be a reason why multifaceted QIPs acting on different barriers in a chain are likely to be more effective than single interventions.

Our research particularly revealed the GPs feelings on collaborative shared care. While some of them disagree on the added value of diabetes educators, many GPs feel some uneasiness regarding the competition with specialist care. These feelings may be reinforced by the typical Belgian healthcare setting, but we believe that they are the expression of a very human nature and thus not unique to the Belgian situation. Literature on this issue, however, is very scarce. Our research also showed that these negative assumptions and feelings can be overcome by paying attention to them and by enhancing the personal contact and communication between the people involved.

The interviews also revealed the limits of a clinician-centered model of patient education and self-management, and confirmed the quantitative results of the study on this issue. Future QIPs could incorporate and test innovative patient-centered methods, like different models on peer support for patients.

Finally, several interviewees reported real concerns on the applicability of the 'traditional' diabetes guidelines in a subset of the patient population, namely the elderly. These concerns have been joined by specific geriatric guidelines. These findings show that quality improvement is not a unidirectional process from guideline to practice. Often, several practitioners express the same difficulties with implementing a guideline. In that case, it might actually reveal a flaw in that guideline rather than a barrier related to the practitioners. And thus QIPs should also be used as instruments to test the feasibility of guidelines as well as to highlight any flaws.

## Competing interests

The authors declare that they have no competing interests.

## Authors' contributions

GG, LB, CVDB and RG participated in the study design and drafted the manuscript. CM, KH and JH participated in the study design and in the discussion of the results. All authors have read and approved the final manuscript.

## Appendix

### Interventions of the quality improvement program

#### Interventions in support of the GP

- Diffusion of a Evidence-based treatment protocol with clear recommendations on:

**1**. Timely follow-up (every three months), with attention to all important parameters (biological risk factors and early signs of complications).

**2**. Global treatment with attention for:

a. Glycaemia control, blood pressure control and blood lipids control.

b. Comprehensive treatment.

i. Healthy lifestyle habits.

ii. Comprehensive drugs treatment including anti-platelet therapy, BP treatment with ACE-inhibition, and statin therapy.

**3**. Target-driven treatment (7% for HbA1c <7%, SBP ≤ 130 mm Hg, LDL-C <100 mg/dl) with treatment intensification whenever the targets are not reached.

**4**. Task description:

a. The GP receives the overall responsibility for the management of diabetes patients. If the GP does not succeed in reaching the targets, he or she can call for help by referring to partners in the diabetes care (interdisciplinary diabetes care team, or IDCT, or hospital-based diabetes clinics).

b. The IDCT functions in support of the GP whenever treatment targets were not reached.

c. The hospital-based diabetes clinic should treat patients with case of complications and with complex insulin therapy schemes.

- **Clinician education and coaching**

a. postgraduate educational sessions on:

i. the evidence-based treatment of T2DM patients, according to the treatment protocol, with special attention to the principles of global cardiovascular treatment and the target driven approach.

ii. the initiation and adjustment of insulin therapy in general practice.

b. Case coaching by the endocrinologist: the GP can call for help by mail or by phone regarding treatment schemes of individual patients without referring them to the specialist.

**- Feedback: **benchmarking feedback: each GP receives feedback on the treatment schemes and on the outcomes of patients of his or her practice in comparison with the results of the entire group.

**- Incentives: **€60 for each included patient; involvement of opinion leaders (endocrinologist from the University Hospital)

#### Interventions in support of the patient

**- Availability of patient education **by a nurse educator, a dietician, or a general internist working together in one IDCT, upon referral by the GP

**- Availability of Home Blood Glucose Material **for patients with insulin therapy initiated by the GP and the IDCT

#### Organizational interventions

**- Team changes: **the IDCT was newly created and acted on the interface between primary and specialist care. The team consisted of a general internist, a diabetes educator (this intervention is innovative in Belgian primary care) and a dietician. It could only be counselled upon referral by the GP and was supervised by the endocrinologist of the hospital-based diabetes clinic and her team trough bi-monthly joint team meetings.

**- Timely data collection: **GPs are asked (by mail and by phone) to deliver diabetes related patient data every three months.

IDCT = Interdisciplinary Diabetes Care Team (endocrinologist, nurse educator, dietician) installed at the primary care level
